# Thyroid cancer survival and prognostic factors in Yantai, China (2012–2022): a population-based study

**DOI:** 10.1186/s12885-024-13211-8

**Published:** 2024-12-30

**Authors:** Haiyun Liu, Shuxia Zhang, Xiaohui Liu, Qianqian Wang, Hongjie Zhang, Weihong Cui

**Affiliations:** 1 Department of Medicine, Shandong College of Traditional Chinese Medicine, Yantai, 264199 China; 2 Yantai Center for Disease Control and Prevention, Yantai, 264003 China; 3 Qixia City People’s Hospital, Yantai, 265300 China; 4 Qingdao Institute of Prevention Medicine, Qingdao Center for Disease Control and Prevention, Qingdao, 266033 China

**Keywords:** Thyroid cancer, Population-based, Overall survival, Prognostic factors

## Abstract

**Background:**

Although thyroid cancer is associated with low mortality rates, significant racial disparities in thyroid cancer outcomes have not been adequately studied in Asia. Moreover, the Asian population consists of different ethnic groups that are not homogeneous. This study aimed to perform a population-based analysis of survival outcomes and prognostic factors in thyroid cancer patients.

**Methods:**

The demographic data and tumor characteristics of all the thyroid cancer patients identified were obtained from the Yantai Cancer Registry. The thyroid cancer-specific death risk in patients was evaluated using the proportion of deaths, standardized mortality ratio (SMR) and absolute excess risk (AER). The Kaplan‒Meier method and the Cox proportional hazards regression model were used to evaluate overall survival (OS) and prognosis.

**Results:**

A total of 10,852 new cases of thyroid cancer occurred with a 5-year OS of 96.20% in Yantai from 2012 to 2022. The SMR decreased from 1.06 (95%CI: 0.93 − 1.33) in 2012 to 0.50 (95%CI: 0.42 − 0.63) in 2022 and the AER decreased from 11.07 (95%CI: -13.42 − 47.39) per 10,000 population in 2012 to -105.02 (95%CI: -149.53 − -63.02) per 10,000 population in 2022. Disparities in the OS of thyroid cancer patients were found across different diagnosis periods, genders, age groups, places of residence, occupational classes, tumor sites and sizes, cervical lymph node metastasis statuses, TgAb levels, pathological types, clinical stages and treatment timings (all *p* < 0.05). Multivariate analysis showed that age group (≥ 65 years: HR = 1.727), tumor site (location in the isthmus: HR = 3.117), tumor size (> 3 cm: HR = 3.170), cervival lymph node metastasis (HR = 1.876), TgAb levels (115 − 500 IU/ml: HR = 7.103; > 500 IU/ml: HR = 13.554), pathological types (MTC: HR = 2.060; ATC: HR = 10.747), clinical stages (stage II: HR = 2.224; stage III: HR = 3.361; stage IV: HR = 3.494), treatment timing (> 3 months: HR = 2.594), diagnosis period (2017 − 2022: HR = 0.633) and gender (female: HR = 0.711) were found to be associated with the risk of death; after stratified adjustment, significant differences in prognostic factors were identified among thyroid cancer patients with varying pathological types.

**Conclusion:**

The risk of death from thyroid cancer in Yantai has significantly decreased and the OS of patients has improved significantly in the past decade. The prognosis of thyroid cancer in this area was notably impacted by various factors and the resolution of survival study outcomes for thyroid cancer patients should be enhanced.

## Introduction

Thyroid cancer, the most common endocrine malignancy, has seen rapidly increasing incidence rates globally since the 1970s, with mortality rates remaining comparatively stable [1], but published data on population-based survival outcomes and prognostic factors are insufficient [2], particularly in East Asia, where previous studies [3–6] have primarily focused on incidence or mortality trends. Despite its low mortality rate [7], significant racial disparities exist in thyroid cancer outcomes [8]. Furthermore, the Asian population comprises diverse ethnic groups that are not homogenous, but racial disparities in outcomes have not been adequately studied [8].

Yantai, located at the junction of the Yellow and Bohai Seas and covering an area of 13,654 km^2^ with a population of 6,535,000 as of 2022, is the eighth largest coastal port city in China and has close social and economic exchanges with neighboring South Korea and Japan. Over the last decade, the incidence of thyroid cancer in Yantai has increased 10.27-fold, with an annual increase of 3.49% by 2022 [9]. This trend is similar to that of South Korea [10], which has the highest thyroid cancer incidence globally [11].

Since 2012, the Yantai Cancer Registry has been established as a national cancer surveillance site in China and was included in the Cancer Incidence Database of Five Continents by the International Agency for Research on Cancer (IARC) in 2023. As a key indicator of the cancer survival status of the whole population, population-based analysis of cancer survival rates can evaluate the progress of cancer control efforts. Therefore, this study aimed to perform a population-based analysis of survival outcomes and prognostic factors in thyroid cancer patients between 2012 and 2022 in Yantai, providing a reference for East Asia and other regions.

## Methods

### Patient data collection

**Fig.1:**
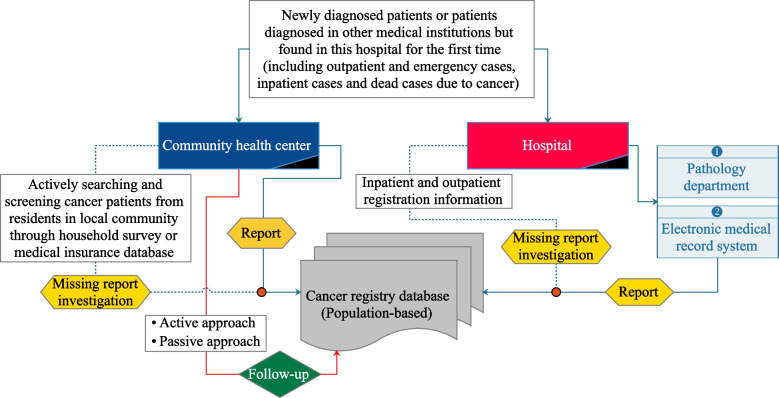
Flowchart of population-based cancer registration and follow-up in Yantai

Patient data were obtained from the Yantai Cancer Registry Center, with cancer information reported by local medical institutions, including hospitals and community health centers. All medical institutions have been conducting regular investigations each year to track patients who fail to report. Community health centers carried out follow-ups to determine the vital status of all registered patients using a combination of active and passive approaches until March 31, 2023. In addition, the Yantai CDC was responsible for the quality control of the data according to Chinese Guideline for Cancer Registration (2016) (Fig. 1). Inclusion criteria were as follows: (1) confirmed by histopathology; (2) the first diagnosis was only one primary thyroid cancer with the ICD-10 code of C73 [12]; (3) follow-up with the definite cause of death; (4) diagnosed between 2012 and 2022. And the exclusion criteria were as follows: (1) lack of complete follow-up information; (2) follow-up less than 2 months. Follow-up period was defined as the length of time from the patient’s diagnosis to the end of follow-up or death.

The prognostic factors analyzed in this study were vital status, diagnosis period, gender, age group, place of residence, occupational class, tumor site and size, cervical lymph node metastasis status, serological parameter, pathological type, clinical stage and treatment method. Of all, age was devided into 3 groups including < 45 years, 45 − 64 years and ≥ 65 years [13, 14]. Occupational class was classified into 4 groups based on the latest version of the International Standard Classification of Occupations (ISCO-08): white-collar class (e.g., managers, professionals, technicians and associate professionals), service class (e.g., clerical, service and sales workers), blue-collar class (e.g., skilled agricultural, forestry and fishery workers, craft and related trades workers, plant and machine operators, assemblers and elementary occupations) and others (e.g., homemakers, students and unemployed workers). Tumor size was classified into 2 groups including ≤ 3 cm and > 3 cm [14]. Serological parameters mainly used anti-thyroglobulin antibodies (TgAb) at the time of diagnosis with the reference range value less than 116 IU/ml [15]. Pathological type was classified into 4 groups based on the criteria of the WHO [16]: papillary thyroid cancer (PTC) with the ICD-O-3 code including 8050, 8260, 8340 − 8344 and 8350; follicular thyroid cancer (FTC) with the ICD-O-3 code including 8330, 8335 and 8339; medullary thyroid cancer (MTC) with the ICD-O-3 code of 8345 and anaplastic thyroid cancer (ATC) with the ICD-O-3 code including 8020 and 8337. Other subtypes (e.g., lymphoma, squamous cell carcinoma and unspecified) were excluded from this study because of their extremely low numbers. The clinical stage was classified following the American Joint Committee on Cancer (AJCC)/TNM risk score from I to IV following the 7th edition scoring [17]. Treatment methods included: (1) therapeutic approaches with the surgery only or surgery combined with radioiodine (RI); (2) delayed surgical treatment following diagnosis was divided into ≤ 3 months and > 3 months according to the treatment timing (The median time from diagnosis to hospital admission for all patients in this study was 3.05 months).

### Statistical analysis

Both SMR and AER were calculated for thyroid cancer-specific death risk, comparing with the East Asian population [18]. The SMR refer to the ratio of observed to expected deaths [19]. The AER was calculated as AER = 10,000 ([number observed-number expected] / [person-years at risk]) [20]. The prognostic factors of patients were presented as numbers (percentages), and the chi-square test was used to assess differences in these qualitative variables. The Kaplan‒Meier method was used to estimate 3- and 5-year overall survival (OS), while the log-rank test was used to compare OS between two or more groups. The Cox proportional hazards regression model was used to calculate the hazard ratio (HR) and associated 95% confidence interval (95% CI) in the univariate and multivariate survival analyses. A multivariate Cox model was carried out using prognostic factors with *p* values < 0.05 in the log-rank test or univariate analysis. In all tests, a *p*-value < 0.05 was considered to indicate statistical significance, and the analyses were performed with R software (version 4.3.2).

## Results

**Fig.2:**
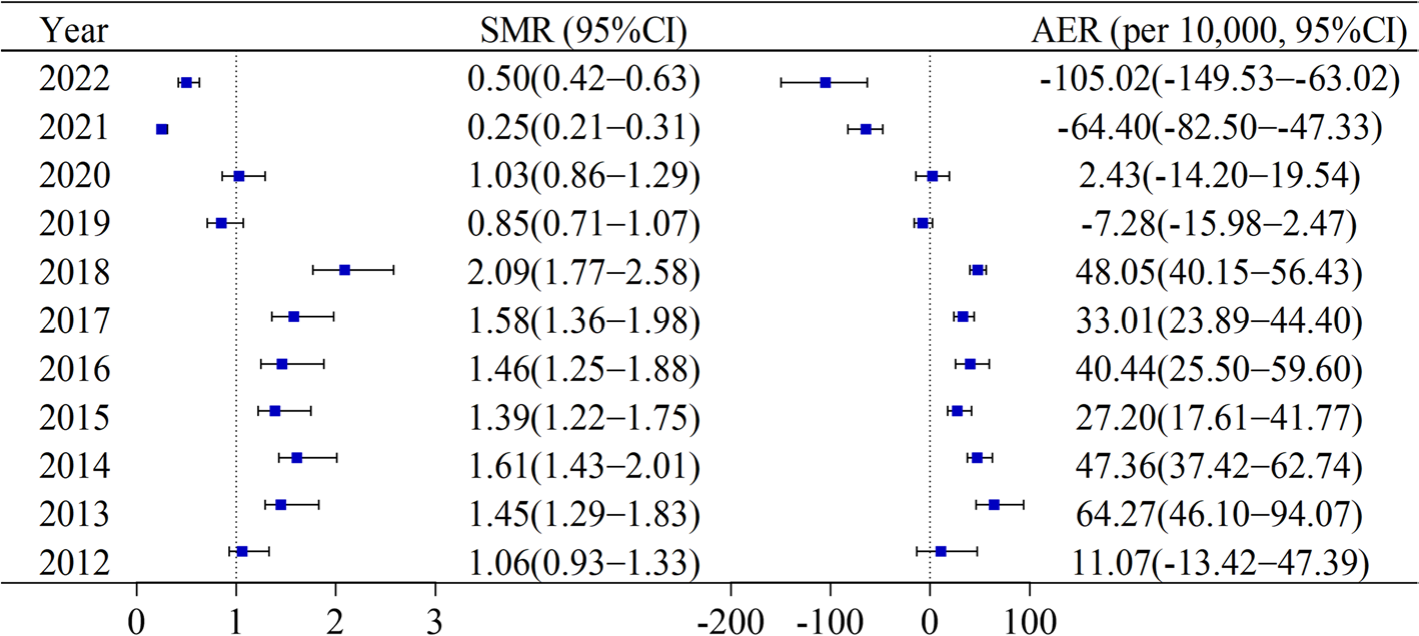
The risk of death among patients with thyroid cancer in Yantai during 2012 − 2022. Note: SMR: Standardized mortality ratio; AER: Absolute excess risk

The total number of 10,852 patients with thyroid cancer was registered in Yantai between 2012 and 2022. Compared with the general population, the risk of death attributable to thyroid cancer showed an overall downward trend year by year with the SMR ranging from 1.06 (95%CI: 0.93 − 1.33) to 0.50 (95%CI: 0.42 − 0.63) and with the AER ranging from 11.07 (95%CI: −13.42 − 47.39) per 10,000 population to −105.02 (−149.53 − −63.02) per 10,000 population from 2012 to 2022 (Fig. 2).

Among all patients between 2012 and 2022, 1,960 were diagnosed in the 2012–2016 period, while 8,892 were diagnosed in the 2017–2022 period, accounting for a significantly larger proportion (81.94%). The majority of patients were female (76.35%), aged 45–64 years (52.52%), living in the urban area (64.74%), in the blue-collar class (56.45%), with unilateral cancer (78.55%), with tumor size ≤ 3 cm (92.19%), with no lymph node metastasis (74.43%), with TgAb < 115 IU/ml (63.39%), with PTC (98.06%), with AJCC stage I (77.83%), through surgery (96.88%) (all *p* < 0.001, Table 1).

**Table 1:** Univariate analysis of prognostic factors in patients with thyroid cancer in Yantai during 2012 − 2022

Characteristics		Death cases (%)	OS (%, 95%CI)		HR (95%CI)	*p*-value
			3-year	5-year		
Diagnosis period	2012 − 2016	201(10.26)	93.98(92.93 − 95.04)	91.78(90.57 − 93.00)	1.000 (reference)	
2017 − 2022	178(2.00)	98.19(97.91 − 98.48)	97.55(97.17 − 97.93)	0.282(0.229 − 0.349)	< 0.001	
Gender	Male	141(5.49)	95.52(94.69 − 96.36)	93.93(92.89 − 94.98)	1.000 (reference)	
	Female	238(2.87)	97.94(97.62 − 98.25)	96.91(96.47 − 97.34)	0.516(0.419 − 0.635)	< 0.001
Age group (years)	< 45	35(0.84)	99.39(99.14 − 99.64)	98.95(98.58 − 99.32)	1.000 (reference)	
	45 − 64	143(2.51)	98.32(97.97 − 98.67)	97.25(96.75 − 97.75)	2.948(2.037 − 4.267)	< 0.001
	≥ 65	201(19.92)	83.78(81.47 − 86.15)	79.51(76.79 − 82.33)	25.657(17.918 − 36.739)	< 0.001
Place of residence	Urban	164(2.33)	98.26(97.95 − 98.58)	97.48(97.06 − 97.90)	1.000 (reference)	
	Rural	215(5.62)	95.72(95.05 − 96.39)	93.83(92.95 − 94.72)	2.475(2.020 − 3.033)	< 0.001
Occupational class	Service class	10(2.30)	98.52(97.35 − 99.71)	98.20(96.88 − 99.54)	1.000 (reference)	
	White-collar class	15(3.16)	97.61(96.22 − 99.02)	96.94(95.28 − 98.63)	1.337(0.601 − 2.976)	0.477
	Blue‐collar class	260(4.24)	96.83(96.38 − 97.28)	95.57(95.00 − 96.14)	1.886(1.003 − 3.547)	0.049
	others	94(2.46)	98.13(97.68 − 98.58)	96.95(96.26 − 97.65)	1.217(0.634 − 2.336)	0.555
Site	Unilateral	291(3.41)	97.56(97.23 − 97.91)	96.25(95.78 − 96.72)	1.000 (reference)	
	Isthmus	42(25.00)	73.60(66.90 − 80.97)	73.60(66.90 − 80.97)	11.425(8.240 − 15.841)	< 0.001
	Multiple	46(2.13)	98.38(97.81 − 98.95)	97.75(97.04 − 98.47)	0.622(0.456 − 0.849)	0.003
Size (cm)	≤ 3	189(1.89)	98.67(98.44 − 98.91)	97.57(97.21 − 97.92)	1.000 (reference)	
	> 3	190(22.41)	50.17(44.71 − 56.31)	48.08(42.63 − 54.22)	3.214(2.615 − 3.949)	< 0.001
Lymph node metastasis	No	75(1.05)	99.63(99.49 − 99.77)	98.62(98.31 − 98.94)	1.000 (reference)	
	Yes	304(8.15)	89.69(88.41 − 90.98)	87.54(85.99 − 89.13)	5.110(2.802 − 13.830)	< 0.001
TgAb (IU/ml)	< 115	37(0.57)	99.63(99.48 − 99.79)	98.71(98.32 − 99.10)	1.000 (reference)	
	115 − 500	75(14.73)	88.76(86.06 − 91.55)	88.76(86.06 − 91.55)	1.143(1.105 − 1.611)	< 0.001
	> 500	267(31.49)	38.39(32.60 − 45.20)	16.67(12.30 − 22.59)	12.872(9.822 − 16.870)	< 0.001
Pathological type	PTC	324(3.04)	97.73(97.43 − 98.02)	96.68(96.29 − 97.08)	1.000 (reference)	
	FTC	14(12.07)	91.75(86.70 − 97.09)	89.22(83.28 − 95.57)	3.615(2.116 − 6.176)	< 0.001
	MTC	19(27.94)	85.18(76.67 − 94.64)	69.78(58.11 − 83.79)	9.971(6.274 − 15.848)	< 0.001
	ATC^*^	22(84.62)	46.15(30.47 − 69.91)	23.08(10.94 − 48.66)	71.125(45.668 − 110.770)	< 0.001
AJCC stage	I	13(0.15)	99.88(99.80 − 99.96)	99.81(99.69 − 99.92)	1.000 (reference)	
	II	80(6.91)	97.18(96.20 − 98.16)	92.98(91.24 − 94.75)	4.041(2.248 − 7.266)	< 0.001
	III	256(21.53)	82.25(80.03 − 84.52)	78.28(75.85 − 80.79)	13.094(7.495 − 22.878)	< 0.001
	IV	30(50.85)	54.38(42.81 − 69.08)	54.38(42.81 − 69.08)	39.519(20.607 − 75.790)	< 0.001
Treatment timing (months)	≤ 3	75(2.11)	98.66(98.41 − 98.81)	97.71(97.35 − 98.08)	1.000 (reference)	
	> 3	114(9.60)	89.90(88.38 − 91.43)	87.55(85.80 − 89.33)	5.287(4.316 − 6.476)	< 0.001
Therapeutic approach	Surgery	304(2.89)	97.36(97.05 − 97.68)	96.20(95.78 − 96.61)	1.000 (reference)	
	Surgery + RI	6(1.77)	98.35(95.52 − 100)	98.35(96.52 − 100)	0.951(0.304 − 2.971)	0.931
All patients		379(3.49)	97.36(97.05 − 97.68)	96.20(95.79 − 96.61)	―	―

*TgAb* Anti-thyroglobulin antibodies, *PTC* Papillary thyroid cancer, *FTC* Follicular thyroid cancer, *MTC* Medullary thyroid cancer, *ATC* Anaplastic thyroid cancer, *RI* Radioiodine

*The longest survival among patients with ATC in this study was 1.98 years, which is less than 2 years, so survival rates at 6 months and 1 year were calculated

**Fig. 3:**
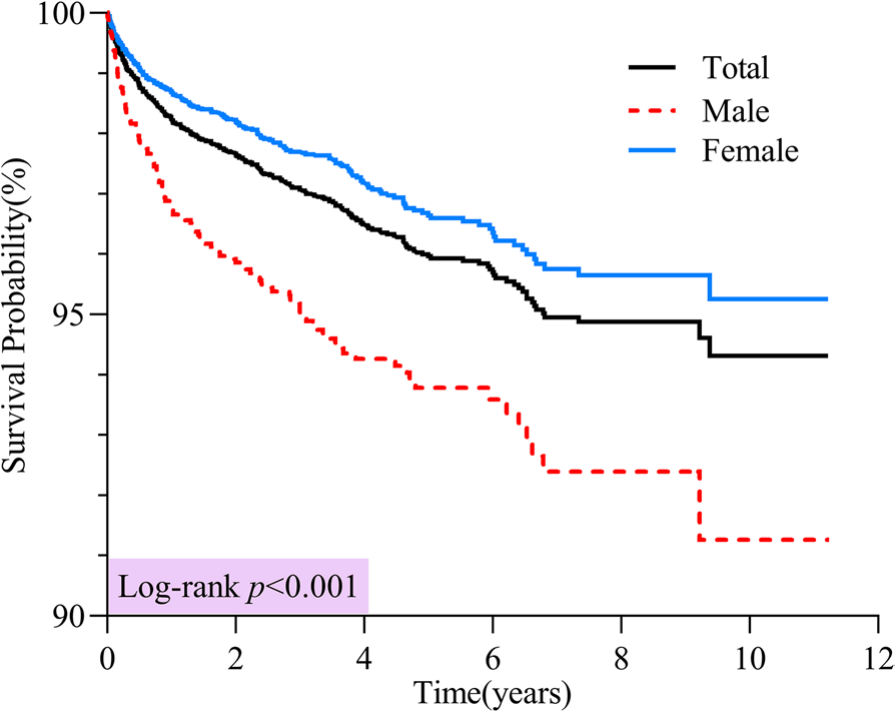
Overall survival of patients with thyroid cancer diagnosed in Yantai during 2012 − 2022

Among the 10,852 patients who were followed up with a median follow-up of 3.39 [1.67 − 5.21] years, 379 patients (3.49%) died due to thyroid cancer, with a median survival time of 0.74 years. Meanwhile, the OS of 97.36% at 3 years and 96.20% at 5 years were found, and men had a lower 5-year OS (93.93%) than women (96.91%) (*p* < 0.001) (Table 1 and Fig. 3); PTC resulted in 324 deaths, accounting for just 3.04% of all PTC patients, and 14 (12.07%) for FTC, 19 (27.94%) for MTC and 22 (84.62%) for ATC. AJCC stage I had 13 deaths accounting for only 0.15%, and 80 (6.91%) for stage II, 256 (21.53%) for stage III and 30 (50.85%) for stage IV (Table 1).

Among all patients, those diagnosed during the period of 2012–2016 (91.78%), living in rural areas (93.83%), aged ≥ 65 years old (79.51%), working in blue-collar occupations (95.57%), with lesions located in the isthmus (73.60%), with tumor size > 3 cm (48.08%), with lymph node metastasis (87.54%), with TgAb > 500 IU/ml (16.67%), with AJCC stage IV (54.38%) and with treatment timing > 3 months after diagnosis (87.55%), had a lower OS (Table 1). Among all pathological types, PTC patients had the highest 5-year OS (96.68%), followed by those with FTC (89.22%) and MTC (69.78%); ATC patients had the lowest OS, with the longest survival among them being only 1.98 years. Additionally, the 1-year OS for ATC patients was 23.08% (Table 1).

**Fig.4:**
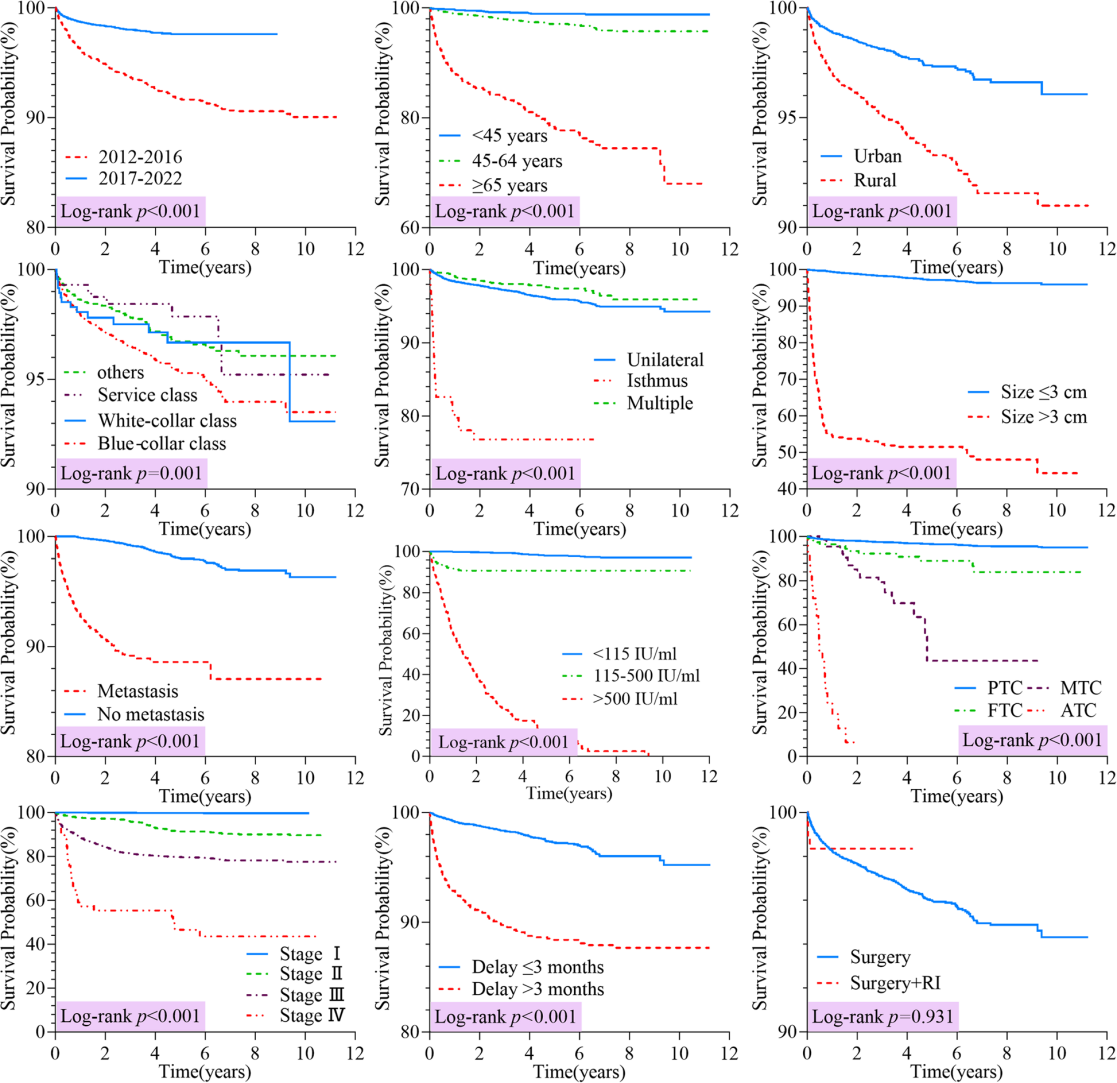
Kaplan–Meier curves comparing the overall survival according to subgroups of prognostic factors. Notes: TgAb: Anti-thyroglobulin antibodies; FTC: Follicular thyroid cancer; MTC: Medullary thyroid cancer; ATC: Anaplastic thyroid cancer; RI: Radioiodine

According to the log-rank test, statistically significant differences in OS were found among different diagnosis periods (*p* < 0.001), age groups (*p* < 0.001), places of residence (*p* < 0.001), occupational classes (*p* = 0.001), tumor sites and sizes (*p* < 0.001), lymph node metastasis statuses (*p* < 0.001), TgAb levels (*p* < 0.001), pathological types (*p* < 0.001), clinical stages (*p* < 0.001) and treatment timings (*p* < 0.001); yet no significant difference was found between the two therapeutic approaches (*p* = 0.931) (Fig. 4).

The survival status of prognostic factors showed significant differences among 4 pathological types (Table 2). In PTC patients, OS showed statistical differences among prognostic factors (*p* < 0.05); in FTC patients, no statistical differences in OS were found among diagnosis periods, genders, places of residence, occupations and treatment timings (*p* > 0.05); in MTC patients, OS showed statistical differences only among tumor sizes, lymph node metastasis statuses, AJCC stages, and treatment timings (*p* < 0.05); in ATC patients, OS showed statistical differences only among occupations, tumor sizes, TgAb levels and lymph node metastasis statuses (*p* < 0.05). Besides, for all pathological types, there were no statistical differences in OS between the two therapeutic approaches (*p* > 0.05).

**Table 2:** Overall survival analysis on patients with thyroid cancer according to pathological types in Yantai during 2012 − 2022

Charateristics		PTC		TC		MTC		ATC*	
		5-year OS (%, 95%CI)	Log-rank(*p*-value)	5-year OS (%, 95%CI)	Log-rank(*p*-value)	5-year OS(%, 95%CI)	Log-rank (*p*-value)	1-year OS (%, 95%CI)	(%, 95%CI)Log-rank (*p*-value)
Diagnosis period	2012 − 2016	92.57(91.40–93.76)	< 0.001	86.36(76.80–97.12)	0.366	60.99(42.59–87.32)	0.222	18.65(9.24–37.91)	0.093
2017 − 2022	97.91(97.56–98.27)	92.63(86.56–99.12)	75.06(61.14–92.14)	32.89(16.57–65.30)					
Gender	Male	94.93(93.96–95.92)	< 0.001	84.92(73.44–98.19)	0.209	61.06(44.29–84.17)	0.051	26.37(10.13–68.69)	0.066
	Female	97.22(96.81–97.64)		91.14(84.47–98.33)		77.20(62.52–95.32)		20.51(6.54–64.29)	
Age group(years)	< 45	99.00(98.63–99.37)	< 0.001	96.77(90.75–100)	< 0.001	75.00(42.59–100)	0.746	1	0.173
	45 − 64	97.70(97.24–98.16)		94.47(88.55–100)		67.99(53.67–86.13)		22.22(6.55–75.44)	
	≥ 65	81.23(78.52–84.04)		66.06(47.75–91.38)		72.54(52.78–99.71)		16.67(4.95–56.09)	
Place of residence	Urban	97.88(97.49–98.27)	< 0.001	91.72(83.09–100)	0.873	70.41(55.67–89.05)	0.616	19.23(5.81–63.67)	0.469
	Rural	94.44(93.58–95.30)		87.12(79.15–95.88)		67.64(49.78–91.90)		26.92(10.46–69.29)	
Occupational class	Service class	98.46(97.24–99.70)	0.001	1	0.674	96.70(30.00–100)	0.459	#	0.037
	White-collar class	97.13(95.51–98.78)		1		89.33(64.19–95.82)		▲	
	Blue‐collar class	96.08(95.53–96.63)		86.87(79.19–95.29)		72.38(57.54–91.05)		32.16(15.99–64.70)	
	others	97.48(96.85–98.11)		90.40(78.18–100)		67.25(49.31–91.71)		▲	
Site	Unilateral	96.74(96.30–97.19)	< 0.001	92.79(87.29–95.63)	0.012	69.16(57.00–83.91)	0.759	22.73(9.91–52.15)	0.227
	Isthmus	74.64(67.90–82.04)		#		#		15.66(7.25–71.14)	
	Multiple	98.11(97.44–98.78)		96.49(95.71–100)		80.00(51.61–100)		50.00(12.50–100)	
Size (cm)	≤ 3	97.79(97.45–98.14)	< 0.001	93.27(88.13–98.71)	< 0.001	75.55(64.15–88.98)	< 0.001	1	0.001
	> 3	53.48(47.73–59.93)		81.44(52.22–85.93)		53.88(38.76–62.28)		5.00(0.74–33.78)	
TgAb (IU/ml)	< 115	98.82(98.44–99.20)	< 0.001	97.03(93.04–100)	< 0.001	88.97(77.77–100)	#	1	0.027
	115-500	90.22(87.64–92.88)		77.78(54.85–100)		#		14.29(2.33–87.69)	
	> 500	19.20(14.19–25.99)		△		#		8.00(1.25–51.36)	
Lymph node metastasis	No	98.91(98.62–99.19)	< 0.001	96.11(91.83–100)	< 0.001	73.05(61.29–87.07)	< 0.001	80.00(51.61–100)	0.002
	Yes	88.67(87.15–90.22)		33.00(12.24–88.99)		▲		6.67(1.04–42.59)	
AJCC stage	I	99.83(99.72–99.94)	< 0.001	95.91(90.42–100)	0.035	1	0.003	#	0.280
	II	93.27(91.53–95.04)		84.28(68.91–100)		92.86(80.30–100)		1	
	III	79.33(76.88–81.87)		79.62(66.30–95.61)		45.84(28.75–73.08)		▲	
	IV	74.27(60.38–91.35)		69.33(59.58–84.39)		50.00(12.50–100)		23.81(10.43–54.36)	
Treatment timing (months)	≤ 3	97.77(97.41–98.14)	< 0.001	92.14(86.17–98.53)	0.101	1	0.006	▲	0.372
	> 3	90.16(88.56–91.80)		76.36(60.13–96.97)		58.81(44.83–77.15)		22.00(10.27–47.11)	
Therapeutic approach	Surgery	96.66(96.26–97.05)	0.094	90.94(85.35–96.90)	0.061	69.16(57.31–83.46)	0.546	25.00(11.84–52.79)	0.060
	Surgery + RI	1		53.33(21.42–100)		1		▲	

*TgAb* Anti-thyroglobulin antibodies, *PTC* Papillary thyroid cancer, *FTC* Follicular thyroid cancer, *MTC* Medullary thyroid cancer, *ATC* Anaplastic thyroid cancer, *RI* Radioiodine

*1-year OS was calculated; All patients survived or were followed up for less than 1 year; All patients survived or were followed up for less than 5 year

**#**Missing observations made the calculation impossible, resulting in a lack of results

**Fig. 5:**
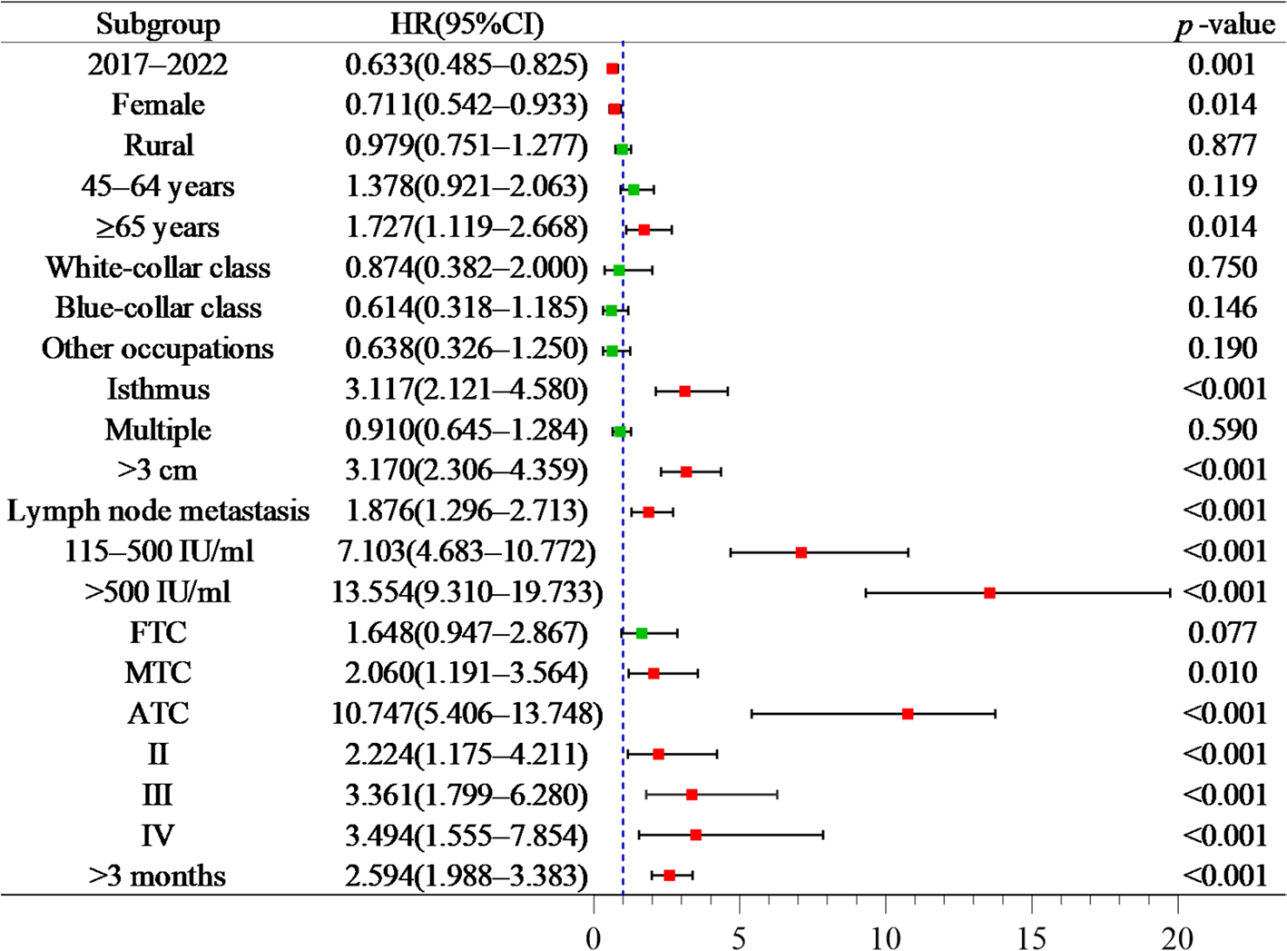
Multivariate analysis on prognosis factors for thyroid cancer survival in Yantai during 2012 − 2022. Notes: FTC: Follicular thyroid cancer; MTC: Medullary thyroid cancer; ATC: Anaplastic thyroid cancer

Multivariate analysis showed that age group (≥ 65 years: HR = 1.727), tumor site (location in the isthmus: HR = 3.117), tumor size (> 3 cm: HR = 3.170), lymph node metastasis (HR = 1.876), TgAb levels (115 − 500 IU/ml: HR = 7.103; > 500 IU/ml: HR = 13.554), pathological types (MTC: HR = 2.060; ATC: HR = 10.747), clinical stages (stage II: HR = 2.224; stage III: HR = 3.361; stage IV: HR = 3.494) and treatment timing (> 3 months: HR = 2.594) significantly increased the risk of death in thyroid cancer patients (*p* < 0.05). Diagnosis period (2017 − 2022: HR = 0.633) and gender (female: HR = 0.711) were found to be associated with a decreased risk of death (*p* < 0.05, Fig. 5).

But after adjustment for stratified analysis by pathological types (Table 3), gender was no longer an independent protective factor, while lymph node metastasis remained an independent risk factor for all pathological types; tumor size was not identified as an independent risk factor for MTC patients. Moreover, diagnosis period and age group were independent prognostic factors only for PTC patients; tumor site and TgAb levels were independent risk factors only for PTC and FTC patients; treatment timing was an independent risk factor only for PTC and MTC patients.

**Table 3:** Multivariate analysis on prognosis factors by pathological types for thyroid cancer survival in Yantai during 2012 − 2022

Subgroup	PTC	FTC	MTC	ATC
	HR(95%CI)	HR(95%CI)	HR(95%CI)	HR(95%CI)
2017 − 2022	0.555(0.410–0.750)	–	–	–
Female	1.193(0.896–1.590)	–	–	–
Rural	1.017(0.759–1.363)	–	–	–
45 − 64 years	1.369(0.901–2.079)	0.694(0.179–2.684)	–	–
≥ 65 years	1.670(1.053–2.650)	3.100(0.834–11.520)	–	–
White-collar class	0.990(0.417–2.349)	–	–	0.408(0.046–3.618)
Blue-collar class	0.624(0.311–1.249)	–	–	0.554(0.184–1.673)
Other occupations	0.678(0.333–1.379)	–	–	0.506(0.125–1.022)
Isthmus	2.824(1.889–4.220)	3.037(1.845–10.430)	–	–
Multiple	0.780(0.535–1.137)	2.014(0.593–6.840)	–	–
> 3 cm	2.924(2.070–4.132)	1.833(1.495–3.955)	2.226(0.594–8.336)	4.539(2.049–5.207)
Lymph node metastasis	2.234(1.475–3.384)	2.168(1.678–8.274)	3.603(2.411–9.587)	4.083(2.233–11.058)
115 − 500 IU/ml	7.325(4.660–11.524)	1.585(0.167–15.050)	–	0.354(0.282–3.971)
> 500 IU/ml	14.693(9.686–22.290)	13.320(1.778–18.977)	–	0.287(0.035–5.562)
II	2.693(1.344–5.396)	1.915(1.402–9.121)	2.538(1.314–3.943)	–
III	3.591(1.811–7.119)	3.721(1.622–14.390)	2.735(1.834–6.228)	–
IV	3.487(1.399–8.693)	4.505(1.460–15.520)	4.762(3.755–8.270)	–
> 3 months	2.596(1.958–3.441)	–	1.779(1.621–3.087)	–

*FTC* Follicular thyroid cancer, *MTC* Medullary thyroid cancer, *ATC* Anaplastic thyroid cancer

–According to the log-rank test, there was no statistically significant difference in OS

## Discussion

Our population-based analysis showed that the 5-year OS of thyroid cancer in Yantai was 96.20%, slightly higher than the 95.30% reported in Asian countries based on a systematic review and meta-analysis [21], but obviously lower than the approximately 100% reported in the United States [22, 23] and South Korea [10] during the same period. Nevertheless, over the past decade, the death risk from thyroid cancer in Yantai has significantly decreased, with the 5-year OS from 2017 to 2022 being 6.29% higher than that from 2012 to 2016.

Many studies [24, 25] have indicated that thyroid cancer mainly occurs in women, likely due to gender differences in health-seeking behavior and screening, with women being more proactive than men. Over the past decade in Yantai, women accounted for 76.35% of thyroid cancer patients, and their survival outcomes were better than those of men. However, after the multivariate stratified analysis by pathological types, the effect of gender was eliminated, and it was no longer an independent prognostic factor, which was entirely consistent with previous studies [2, 26–29] on prognostic factors of both well-differentiated and anaplastic thyroid cancers.

Univariate analysis showed that OS decreased with age. Patients under 65 years old, who accounted for more than 90% of total patients, maintained a 5-year OS above 95%, significantly higher than those over 65 years old, suggesting that the excellent prognosis of thyroid cancer in Yantai was not easily affected by the aging population. However, this age effect was specific to PTC patients, which was consistent with many existing studies [2, 14, 26–28, 30]. Currently, the cut-off age was recognized as a prognostic factor only for differentiated thyroid cancer [31], yet research on its role in the prognosis of both MTC and ATC patients remained scarce [32–34].

We found that among all patients with differentiated thyroid cancer, although the proportion of those with lesions located in the isthmus is the smallest, these patients have the highest death risk and the lowest 5-year OS. In contrast, the pathological types of MTC and ATC seemed not sensitive to the tumor site. This aligned with the findings of Campenni [35], who indicated that thyroid cancer in the isthmus tends to behave more aggressively and has a worse prognosis than those originating in the glandular lobe. Several studies [36–38] have reported that patients with thyroid cancer in the isthmus exhibited higher rates of lymph node metastasis, indicating a poor prognosis. Our data showed that among patients with isthmus-located lesions, the proportion of lymph node metastasis reached 40%, and their 5-year OS was significantly lower than those without metastasis. Additionally, we found that lymph node metastasis was an effective prognostic factor for patients with all pathological types.

Considering that TgAb interferes with the accuracy of thyroglobulin (Tg) measurement [39, 40] and the issue of multicollinearity between TgAb and Tg in multivariate analysis, only TgAb was included into our regression model. The results showed that patients with higher TgAb level had poorer survival outcomes and was an independent risk factor for differentiated thyroid cancer, consistent with the previous study [41].

In general, the mortality in patients with stage IV and ATC was high [42]. We found a wide variation in survival rates depending on the pathological type. PTC patients had the highest 5-year OS, which reached more than 95%; patients with FTC and MTC had a better prognosis but worse outcomes than those with PTC; unlike differentiated thyroid cancer, ATC patients had the lowest OS with the longest survival time of only 1.98 years in our study. Although ATC accounted for less than 1% of our patients, it was estimated to be the main cause of thyroid cancer-related mortality. We also showed that ATC patients faced a risk of death over 71 times higher than that of other patients, and even after adjusting for other factors, the risk was still over 10 times higher, suggesting that ATC remains one of the most aggressive and fatal solid tumors. A notable disparity in survival was also identified among different AJCC stages. Patients with Stage IV had the lowest 5-year OS of 54.38%, whereas survival rates for those before Stage III were higher than 90%, which indicated that early detection of thyroid cancer has almost no impact on survival. Similar results [2, 14, 28] have previously been described and our study reinforced the importance of early-stage thyroid cancer screening in improving the survival of patients. However, stratified adjustments showed no significant impact of AJCC stage on survival or risk of death in ATC patients. Contrary to our finding, certain studies [27, 29, 43] showed that the clinical stage was an independent prognostic factor for ATC patients.

The association of delayed surgical treatment with clinical outcomes remains a subject of ongoing controversy [44]. Our study showed that immediate surgery after diagnosis significantly improved survival in PTC and MTC patients, whereas the risk of death rose significantly for those undergoing surgery over 3 months after diagnosis. This was similar to the findings of Fligor [45], who analyzed the OS of 103,812 PTC patients using the National Cancer Database from 2004 to 2016 and found that increasing time to surgery was a significant prognostic factor and delaying by 3 months led to a 1.30-fold increase in the risk of death. However, our study found no difference in the impact of delayed surgery on the survival prognosis of FTC and ATC patients. Despite this, this finding did not provide definitive evidence for intentionally delayed surgery, due to the small number of such patients and the lack of specified reasons for treatment delays in our data. The optimal treatment timing for thyroid cancer surgery following diagnosis still requires in-depth research, but it also raises serious medical ethical issues.

No effect of radioiodine therapy on prognosis was found in our study, which was consistent with some researchers [29, 31, 46] who failed to demonstrate a survival benefit of radioiodine therapy. However, due to the high medical costs associated with radioiodine therapy, along with the low hospitalization reimbursement ratio and the inability to claim reimbursement for outpatient services under Yantai's medical insurance policies [47], the frequency of patients receiving radioiodine therapy in our data is too low, which may have limited the statistical power to valuate the prognostic value. But a previous study [48] has reported that radiotherapy could provide benefit in terms of on loco-regional control but was not associated with an increase in survival. Moreover, as found in a previous study [14], tumor size over 3 cm was an important variable affecting patient prognosis.

The limitation of this study was that collecting high-resolution tumor biological registry and follow-up data through cancer registries in China was often challenging [49], which might introduce potential bias into our multivariate analysis, but this study still had significant reference value for assessing the prognosis of thyroid cancer patients with different pathological types in the East Asian region.

## Conclusions

The survival rate of thyroid cancer in Yantai has improved significantly in the past decade and was comparable to the average of Asian countries at present. The prognosis of thyroid cancer patients in this area was generally affected by a variety of complex factors, including age, tumor site and size, cervical lymph node metastasis status, serological parameter, pathological type, clinical stage and timing of treatment. Further research is necessary to enhance the resolution of survival study outcomes.

## Data Availability

The anonymised data collected are available as open data on the figshare website. 10.6084/m9.figshare.27693300.
